# Randomized or real-world? Integrating imaging evidence for clinical practice

**DOI:** 10.1093/ehjimp/qyaf131

**Published:** 2025-10-25

**Authors:** Paolo Frumento, Alessia Gimelli

**Affiliations:** Department of Political Sciences, University of Pisa, via Serafini 3, 56126 Pisa, Italy; Imaging Department, Fondazione Toscana G. Monasterio, Pisa, Italy

## Introduction

Cardiovascular imaging has become an indispensable pillar of modern cardiology, shaping clinical decision-making across prevention, diagnosis, and therapy. From the early detection of subclinical disease to the stratification of ischemic risk, from monitoring treatment response to guiding interventions, imaging is no longer ancillary but central to patient care. With such a pervasive role, the key question is no longer whether imaging influences outcomes, but how robust and reliable the evidence supporting its use truly is.

To address this question, we selected three landmark studies that represent complementary but distinct methodological paradigms: *SCOT-HEART*^1^ [a randomized trial evaluating anatomical imaging with coronary CT angiography (CTA)], *ISCHEMIA*^2^ (a randomized comparison of invasive vs. conservative management guided by functional testing), and *EURECA*^3^ (a large prospective registry assessing adherence to guideline-recommended diagnostic strategies in real-world settings). These studies differ in enrollment criteria, population size, and analytic approach, providing an opportunity for a comparative analysis of how statistical design influences interpretation and clinical translation in cardiovascular imaging.

Understanding the differences between randomized controlled trials and observational studies is therefore essential. Both approaches contribute in complementary but distinct ways to the scientific foundation of imaging. Randomized trials,^4^ through the principle of randomization, minimize bias and provide the highest level of internal validity, allowing causal inference. They test whether imaging-guided strategies improve hard clinical outcomes such as death, myocardial infarction, or heart failure hospitalization. Observational studies,^5^ whether prospective registries or analyses of real-world data, extend this evidence to broader and more heterogeneous populations, offering insights into generalizability, adherence to guidelines, safety, and barriers to implementation in everyday clinical settings.

In cardiovascular imaging, this distinction carries particular weight because the ‘intervention’ is not a drug or device, but a diagnostic strategy embedded in a broader clinical pathway. Trials rarely compare ‘imaging vs. no imaging.’ Instead, they typically evaluate different diagnostic approaches—such as coronary CTA vs. functional testing—or assess whether management informed by imaging translates into improved outcomes. In this way, randomized trials provide high-certainty answers to narrow, carefully defined questions, while observational registries capture the more complex and heterogeneous reality of daily practice.

Several landmark studies illustrate this interplay. The *SCOT-HEART*^1^ trial randomized 4146 patients with stable chest pain to standard care or standard care plus coronary CTA, using a minimization algorithm to balance baseline risk factors. Analyses followed an intention-to-treat principle, with time-to-event comparisons by Cox proportional hazards models adjusted for center and baseline covariates. The pre-specified long-term primary endpoint—death from coronary heart disease or non-fatal myocardial infarction—was reduced at 5 years (hazard ratio 0.59; 95% CI 0.41–0.84; *P* = 0.004). The trial was powered for an absolute risk reduction of 2.8% and reached its target with 80% power, confirming the prognostic impact of early anatomical imaging.

In contrast, the *ISCHEMIA* trial^2^ enrolled 5179 patients with moderate-to-severe ischemia on non-invasive testing to evaluate whether an initial invasive strategy improved outcomes over conservative management. The primary composite endpoint (cardiovascular death, myocardial infarction, hospitalization for unstable angina, heart failure, or resuscitated cardiac arrest) was analyzed with Cox regression, but the proportional hazards assumption was violated. Investigators therefore complemented standard models with non-parametric cumulative incidence analyses, restricted mean event-free time, and Bayesian sensitivity analyses. These revealed that early procedural events offset later benefit, highlighting the importance of time-dependent effects and modeling assumptions.

Complementing these RCTs, the *EURECA*^3^ registry offered a real-world perspective. Including 5156 patients from 73 European centers, it examined adherence to the 2019 ESC guidelines for chronic coronary syndromes. Logistic regression with backward selection identified predictors of compliance with imaging-based recommendations, and sensitivity analyses—excluding patients with prior CAD and comparing pre/post-COVID-19 periods—confirmed robustness. Goodness-of-fit was verified with the Hosmer–Lemeshow test. *EURECA* demonstrated that adherence to imaging-guided diagnostic algorithms was associated with higher diagnostic yield and more appropriate revascularization.

Together, these studies exemplify how randomized and observational evidence complement one another. *SCOT-HEART*^1^ and *ISCHEMIA*^2^ highlight the statistical rigor and causal inference possible through randomization, while *EURECA*^3^ reflects the external validity and implementation challenges of imaging in everyday care. Their methodological differences—power estimation, handling of non-proportional hazards, and management of confounding—underscore the need for transparency and appropriate statistical design in imaging research.

## Methodological and statistical challenges

Randomized trials and observational registries face distinct methodological limitations. Randomized trials provide strong internal validity but require large sample sizes to account for variability in imaging measurements. They are costly, time-consuming, and risk being outdated by the time results are published, given the pace of technological change. Moreover, they usually test diagnostic strategies rather than imaging *per se*.

For instance, *SCOT-HEART*^1^ used an *a priori* power calculation to detect a modest absolute risk reduction, while *ISCHEMIA*^2^ revised its sample size mid-trial to preserve statistical power as event rates declined. Such adaptations illustrate the challenge of designing imaging trials around dynamic technologies and uncertain baseline risks.

Observational studies, such as *EURECA*,^3^ include broader and more heterogeneous populations but are vulnerable to confounding and selection bias. Robust analytic techniques—multivariable logistic regression, propensity score adjustment, or target trial emulation—can mitigate but not eliminate these limitations. Variability in image acquisition, interpretation, and reporting further complicate inference. Transparent reporting of these factors is crucial for interpreting external validity.

Both designs are also exposed to challenges of power estimation and data heterogeneity. Variability across centers, inter- and intra-observer differences, and low event rates frequently lead to underpowered analyses if not properly anticipated. Statistical transparency—particularly in handling missing data, verifying assumptions, and adjusting for multiple testing—is essential for credible conclusions.

## Clinical pros and cons of each design

Randomized controlled trials remain the standard for demonstrating the efficacy of imaging-guided strategies. Randomization minimizes bias and allows causal relationships between diagnostic pathways and clinical outcomes to be established. This explains why RCTs carry decisive weight in clinical guidelines. However, most imaging RCTs are explanatory rather than pragmatic, often optimizing internal validity at the expense of generalizability. They are resource-intensive, lengthy, and vulnerable to technological obsolescence by the time results are published. Pragmatic designs, adaptive randomization, and registry-based randomized trials represent promising approaches to bridge the gap between controlled and real-world evidence. Importantly, RCTs seldom isolate the ‘effect’ of imaging itself; instead, they assess entire diagnostic strategies integrated within complex care pathways.

Observational studies address these limitations by reflecting real-world practice and population diversity. Large registries capture how imaging is implemented across various healthcare systems, providing essential data on adherence, safety, and variability. Their main weakness is the absence of randomization, which makes results inherently susceptible to residual confounding—even when advanced adjustments are applied. Modern analytic techniques, including marginal structural models and causal machine learning, allow for better correction for time-dependent confounding and selection bias, expanding the inferential capacity of observational imaging research. EURECA exemplifies both the strengths and the constraints of this design: it quantifies real-world implementation but cannot alone establish causality.

Ultimately, the two designs answer different but equally important questions. Randomized trials establish causal efficacy under controlled conditions, while observational studies assess applicability, sustainability, and health-system impact in everyday care. Only through integration of both can we fully characterize the real-world clinical value of cardiovascular imaging.

## Lessons from trials and registries

The literature on cardiovascular imaging provides multiple examples of this complementarity. Randomized strategy trials have shown that imaging can improve diagnostic certainty, influence therapeutic decisions, and occasionally reduce downstream events—but also that not every imaging-defined risk marker translates into improved outcomes. ISCHEMIA exemplified this, showing that even severe ischemia did not justify invasive revascularization over optimized medical therapy in terms of survival.

Large observational registries reveal how imaging is actually practiced, exposing variability in indications, adherence to guidelines, and the uptake of quantitative biomarkers. EURECA confirmed that adherence to evidence-based diagnostic algorithms enhances diagnostic yield and procedural appropriateness. Studies based on PET and CT further underline the prognostic and pathophysiologic value of quantitative imaging markers—information often beyond the practical reach of RCTs.

Together, these studies demonstrate that imaging evidence evolves along a continuum—from tightly controlled efficacy testing (SCOT-HEART, ISCHEMIA) to pragmatic implementation and health-system evaluation (EURECA). Randomized studies define causal effects under ideal conditions, while observational data reveal how these effects unfold across diverse populations and care settings. Both are indispensable to understanding the true impact of imaging on patient outcomes.

## Conclusions

Cardiovascular imaging requires a hybrid evidence model that integrates randomized and real-world data. Randomized controlled trials remain essential for causal inference, while large registries and real-world analyses provide scale, generalizability, and implementation insight.

The value of imaging research ultimately depends on convergence toward clinically meaningful outcomes. Harmonizing imaging variables across modalities and centers, ensuring transparent reporting, and linking data through federated analyses, trial emulation, and adaptive frameworks will produce evidence that is both statistically rigorous and clinically relevant. Only through this balanced approach can cardiovascular imaging continue to inform and improve patient care in real-world practice.

## Data Availability

No new data were generated or analysed in support of this research.
